# Flexural Behavior of Full-Scale Damaged Hollow RC Beams Strengthened with Prestressed SCFRP Plate under Four-Point Bending

**DOI:** 10.3390/polym14142939

**Published:** 2022-07-20

**Authors:** Baojun Li, Lingkai Zeng, Xinyan Guo, Yilin Wang, Zhiheng Deng

**Affiliations:** 1 College of Civil Engineering and Architecture, Guangxi University, Nanning 530004, China; jamberly@163.com (B.L.); dengzh@gxu.edu.cn (Z.D.); 2 Guangxi Transportation Science and Technology Group Co., Ltd., Nanning 530001, China; 3 School of Civil Engineering and Transportation, South China University of Technology, Guangzhou 510640, China; ctlkzeng@mail.scut.edu.cn; 4 Magnel–Vandepitte Laboratory, Department of Structural Engineering and Building Materials, Ghent University, Technologiepark—Zwijnaarde 60, 9052 Ghent, Belgium; yilin.wang@ugent.be

**Keywords:** prestress, steel-carbon fiber reinforced polymer (SCFRP), damaged, hollow RC beam, full-scale

## Abstract

The advantages of using prestressed carbon fiber reinforced polymer (CFRP) for strengthening and retrofitting structures have been reported in recent years. In this regard, most of the studies on prestressed CFRP technique have been carried out in the laboratory test with small-scale and no damage (reinforced concrete) RC beam. However, the real structures that need to be retrofitted in service are often degraded or damaged due to early cracking. This paper aims at studying the effect of prestressed CFRP method on full-scale and damaged RC beams. The damaged levels of four full-scale damaged hollow RC beams taken from an old bridge were evaluated. One damaged beam was tested to check the residual capacity, and the other three were strengthened with prestressed composite strengthened CFRP and steel-carbon fiber reinforced polymer (SCFRP). The flexural behavior of non-strengthened and prestressed strengthened beams was investigated. During the experiments, the failure modes, deflection, yield and ultimate load, strains of concrete, steel reinforcements, and SCFRP were measured and analyzed. The results showed that the stiffness at the elastic stage was increased by 64.9%, 66.9%, and 67.1% after strengthened by SCFRP with 30%, 40%, and 60% prestressing level. Moreover, the ultimate load of damaged hollow RC beams were improved by 19.53%, 21.82%, and 31.9%, respectively. The flexural behavior of the severely damaged RC beam with strength reduction coefficient of 0.65 can be recovered after being strengthened by SCFRP with 40% prestressing levels. Meanwhile, SCFRP-concrete interface debonding failure occurred when the prestressing level exceed 60%, and the characteristics of brittle failure became more evident with increased prestressing level of the SCFRP.

## 1. Introduction

Due to overload, heavy traffic, critical environment, and other adverse factors, the performance of some bridge structures degrades significantly. The cracking, peeling, and sudden collapse of bridge structures bring serious hidden danger to human lives and inestimable economic losses. Reinforcement is a good way to ensure the safety of deficient bridge structures. Furthermore, the prestressing technique applied in reinforcement is an active strengthening method, which can produce negative moment and improves the reinforcement effects effectively.

Carbon fiber reinforced polymer (CFRP) is a type of material with advantage of high strength, lightweightedness, and non-corrosiveness. Nowadays, as a fine prestressing material, more and more CFRP have been widely used for strengthening fields [1,2,3,4,5,6,7]. A large number of experimental and theoretical studies have found that prestressing CFRP can efficiently utilize the tensile capacity of material. Moreover, the cracking loading and ultimate capabilities of strengthened structures enhance significantly [8,9,10,11]. 

Externally bonded reinforcement (EBR) is a strengthening technique, which uses strong epoxy adhesive to attach prestressed CFRP sheets or plate to the surface of strengthened concrete (RC) beam in the tensile area. Garden and Hollaway [12] carried the test in laboratory and bonded the non-prestressed and prestressed CFRP plates on the bottom of rectangular RC beam of 1.0 and 4.5 m lengths, respectively. The prestress level varied from 25% to 50% of the tensile strength. It can be found from the test results that the failure mode of RC beams strengthened with non-prestressed failed due to the separation of plate, while most of the prestressed beam failed by plate fracture. The maximum loads increased by 240% and 168% after strengthened by 50% prestressed level of CFRP. Reza Aram et al. [13] constructed two RC beams of 2.4 m long strengthened by prestressed CFRP with two prestressing levels (18%, 36%) and investigated the flexural behavior. For cracking behavior, three RC beams of 3.6 m long strengthened by prestressed CFRP sheets were tested by Kim [14,15]. The experimental and analytical studies showed less localized damage occurred in reinforced beams and prestress level had great effect on crack behavior of reinforced RC beams. Approximately the 10–20% of the ultimate design strain can meet the ductility recommendation of the standards. Woo [16] considered the bond-slip model of CFRP-concrete interface in finite element analysis to investigate the flexural behavior of prestressed CFRP strengthened RC beam. Moreover, static test of prestressed RC beam with 2.98 m length were carried out in laboratory, it was found that the cracking, yield and ultimate loads of strengthened RC beam were increased by prestressed CFRP. At the maximum load, the ratio of load increased 52.6%.

As can be seen from the above research, most of the study on prestressed CFRP technique have been carried out with small-scale RC beam in laboratory. The field or full-scale applications of prestressed CFRP for strengthening are very limited. Gardern [17] utilized two damaged beams with 18.0 m length from a deteriorated bridge and strengthened them by prestressed CFRP with 30% of ultimate strength. Compared to the unreinforced beams, the load-carrying capacity of strengthened beam was up to 60%. Andra K et al. [18] carried out practical application of prestressed CFRP laminate with 1000 MPa for strengthening Lauter Bridge. Another field application of prestressing CFRP was the strengthening of a metal bridge with two spans of 7.8 m [19], and the ultimate load improved from 7.5 to 40 t. Kim [20] performed repair work using 21% prestresseing level of CFRP sheets to strengthen a 40-year-old damaged concrete beam bridge. Moreover, the corresponding finite element simulation was carried out to analyze the behavior of the bridge before and after reinforcement. The results showed the damaged bridge beam can be restored to an undamaged condition, that proved the effectiveness and availability of prestressed CFRP technique.

The earlier studies on static flexural behavior of RC beam strengthened with prestressed CFRP mainly focused on specimens with no damage. However, the real structures that need to be retrofitted in service are often degraded or damaged due to early cracking. Using CFRP to strengthen RC beam with pre-damage attracted the attention of many researchers in these days. Xie [21] replaced V-notch of tension concrete with polymer mortar and bonded CFRP on degraded RC beams of 1.4 m length by EBR method. The results showed that retrofit method was suitable for RC beams with less than 15% of damage. External bonded CFRP could not work for the damaged beams which undertook more than 50% mass loss of tensile steel. Rabinovitch [22] studied the behavior of cracked RC beams strengthened with non-prestressed and prestressed FRP by theoretical and experimental analysis. The result revealed that prestressing of the FRP strip must be properly anchored to exploit its advantages. Wang [23], Masoud [24] carried out tests to study the behavior of pre-damage RC beam strengthened with CFRP. The experimental results show that the state of corrosion of the steel, the water/cement ratio of the concrete material, and the arrangement and the number of FRP patches all affect the strength as well as the failure mechanisms of retrofitted RC beams. However, they mainly focused on non-prestressed CFRP repaired method, and there were very limited studies that involved the prestressing technique in strengthening damaged RC beams.

Although prestressing CFRP technique is effective for strengthening structures, the field or full-scale applications of prestressed CFRP for strengthening damaged bridge structures are still very limited, especially damaged hollow slab beam. Because the concrete at the bottom of hollow RC beam was thin, the location of the anchorage to stretch the prestressed CFRP was very limited. In this paper, several full-scale damaged hollow slab beams from an old bridge were divided into two groups. One damaged beam in the first group was tested to check the residual capacity, the other three damaged beams in the second group were strengthened by prestressed CFRP and steel-carbon fiber reinforce polymer (SCFRP) plate, respectively. Through the static test, the behavior of prestressed CFRP reinforced hollow slab beams were studied. From the research, the effectiveness of strengthened damaged hollow slab RC beam strengthened with different prestressing level of SCFRP were checked. Moreover, the influence of different damage levels on strengthening effect was discussed.

## 2. Damaged Hollow RC Beams

This chapter describes the details of damaged hollow RC beams. Before strengthening, the analysis of capacity and damage evaluation of damaged hollow RC beams is necessary.

### 2.1. Description of Old Bridge and Damaged Hollow RC Beam

**Figure 1:**
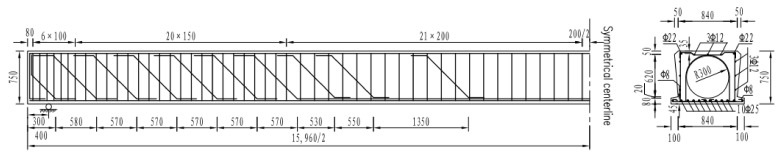
Geometry and reinforcement of the damaged beam (all the dimensions are in mm).

The damaged bridge was built in Sanjiang county, Guangxi Province, China in 2002. As a result of fluvial scouring and heavy traffic, some visual cracks were observed to be between 0.2 mm and 2.5 mm in hollow RC beams. In view of the fact that the low capacity of hollow RC beam does not satisfy the heavy traffic, a new bridge has been constructed to replace the old bridge. As an experiment of full-scale damaged beam reinforcement, four of hollow RC beams with different damage levels were taken from that old bridge. The geometric dimension of the hollow RC beam was 15.96 m long, 1.04 m wide, and 0.75 m high. Moreover, the design concrete grade is C50, and the compressive and tensile strength were 52.5 MPa and 1.89 MPa, respectively. The yield strength and ultimate strength of steel reinforcement are 335 MPa and 510 MPa, respectively. The diameter of the main bearing reinforcement is 25 mm. The typical cross-section dimension and the arrangement of steel reinforcement are shown in Figure 1. 

### 2.2. Capacity of Hollow RC Beam without Damage

**Figure 2:**
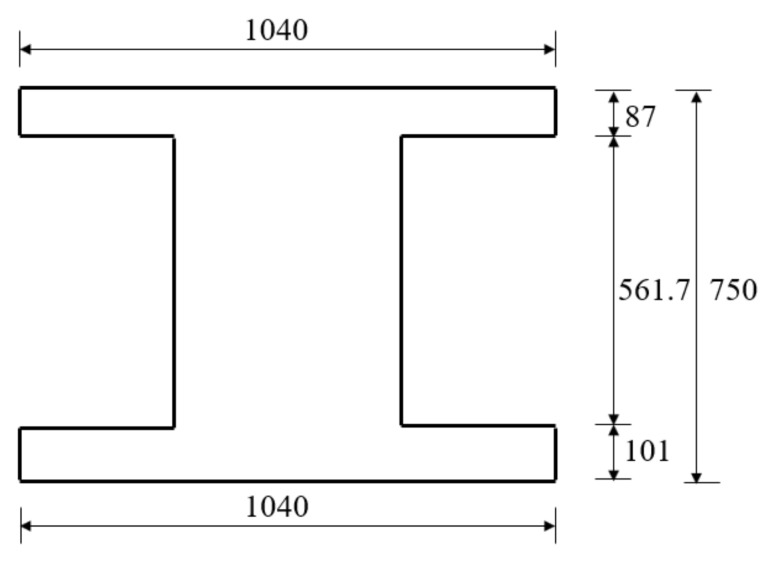
Equivalent cross section (all the dimensions are in mm).

The hollow RC beam is a flexural member in real service and its section bearing capacity can be performed according to the RC structure code. In the calculation process, the hollow section is usually equivalent to I-section, and then the capacity can be calculated. Moreover, the equivalent principle is that the two sections have the same area and moment of inertia as possible. After equivalent transformation, the areas of hollow section and equivalent section are 3977.57 cm^2^ and 3977.55 cm^2^, respectively. Meanwhile, the inertia moments are 2,647,488.32 cm^4^ and 2,647,480.44 cm^4^, respectively. The geometrical characteristic of hollow section and equivalent I-section are shown in Figure 2.

The neutral axis height *x* of T section can be obtained by

(1) $$x=\frac{f_{\mathrm{sd}}A_{s}}{f_{\mathrm{cd}}b_{f}^{\prime }}$$

where *f*_cd_ is the axial compressive strength of concrete, *b*’*_f_* is the upper flange width on I-section, *f*_sd_ is the tensile strength of steel reinforcement, and *A*s is the cover thickness. 

Substituting all the parameters into equation (1), the neutral axis height *x* is 59.0 mm. Meanwhile, concrete above the neutral axis in the flange is subjected to compressive forces, and the main reinforcement and concrete below are subjected to tensile forces. 

Then the flexural load capacity of hollow RC beam is

(2) $$M_{u}=f_{\mathrm{sd}}b_{f}^{\prime }x\left(h_{0}-\frac{1}{2}x\right)$$

where *h*_0_ is the depth of compression zone.

### 2.3. Damage Evaluation 

Based on the specification for inspection and evaluation of load-bearing capacity for highway bridges [25], the bearing capacity of damaged RC structure in ultimate state is obtained by

(3) $$F_{R}\leq \alpha F$$

where *F*_R_ is the residual load, *F* is the ultimate load of RC structures without damage, and α denotes the strength reduction coefficient. 

The parameter α is related to the cross-section reduction factor *R*, structural detection factor *Z*, and deterioration factor $\zeta_{e}$, which can be expressed by

(4) $$\alpha =R\cdot Z\cdot \left(1-\zeta_{e}\right)$$

#### 2.3.1. Cross-Section Reduction Factor R

According to the test of material behavior in three states, including material weathering, carbonization, physical and chemical damage, the cross-section reduction factor *R* is determined by [25]

(5) $$R=\sum_{i=1}^{3}R_{i}\cdot r_{i}$$

where, *R_i_* and $r_{i}$ are the evaluation level and weight coefficient for each of the damage states, respectively. The evaluation level *R_i_* ranges from 1 to 5, indicating that the damage ranges from mild to severe. For concrete structure, the weight coefficients constructure $r_{i}$ are set to 0.1 for material weathering, 0.35 for material carbonization, and 0.55 for physical and chemical damage respectively.

#### 2.3.2. Structural Detection Factor Z

For RC concrete bending structure, structural detection factor *Z* has relevance to structural damage level *D*, which can be expressed as follows [25]:

(6) Z=−0.09D+1.26

where *D* is determined by three damage conditions, such as structural defect, material strength, and natural frequency of vibration of RC concrete structure

(7) $$D=\sum_{i=1}^{3}D_{i}\cdot d_{i}$$

where, *D_i_* and $d_{i}$ are evaluation level and weight coefficient for each of the damage condition, respectively. The evaluation level *D_i_* is similar to *R_i_*, ranging from 1 to 5, indicating that the damage ranges from mild to severe. For concrete structure, the weight coefficient $d_{i}$ is set 0.4 for structural defect, 0.3 for material strength, and 0.3 for natural frequency of vibration.

#### 2.3.3. Deterioration Factor $\zeta_{e}$

The effect of the environment on structural deterioration is denoted by deterioration factor $\zeta_{e}$, which is determined by the deterioration level *K* and environment condition [25]. *K* has relationship with seven conditions, such as structural defect, steel reinforcement corrosion, concrete resistivity, concrete carbonization, depth of steel reinforcement cover, the content of chloride ions, and strength of concrete. The expression of *K* is

(8) $$K=\sum_{i=1}^{7}K_{i}\cdot k_{i}$$

where, *K_i_* and $k_{i}$ are the evaluation level and weight coefficient for each of damage condition, respectively. Moreover, the evaluation level *K_i_* ranges from 1 to 5, which is similar to *R_i_* and *D_i_*. $k_{i}$ are the weight coefficients and set to 0.32 for structural defect, 0.11 for steel reinforcement corrosion, 0.05 for concrete resistivity, 0.2 for concrete carbonization, 0.12 for depth of steel reinforcement cover, 0.15 for the content of chloride ions, and 0.05 for strength of concrete.

From the site survey and examination, the main damage evaluation factors of four damaged RC beams and the flexural load capacity, labelled from A1 to A4, are listed in Table 1.

## 3. Experimental Program 

The four damaged RC beams were divided into two groups, specimen A1 was set as the validate beam without CFRP, to verify the theoretical analysis mentioned above. The other three damaged RC beams were strengthened with prestressed CFRP plates as field application. After strengthening, the names of the specimens A2, A3, and A4 were changed to A2P30, A3P40, and A4P60 for different prestress level (30%, 40%, and 60% of SCFRP ultimate strength). The static tests of four RC beams (A1, A2P30, A3P40, and A4P60) were carried out, and prestressing effects on stiffness, cracking load, yield load, deflection, and capacity of strengthened RC beam were investigated. 

### 3.1. SCFRP Plate

CFRP is a type of material with advantage of high strength, lightweight, and non-corrosive. Nowadays, as prestressing materials, more and more CFRP have been widely used for bridge strengthening. Most of the CFRP plate used in engineering are usually made of CFRP silk and epoxy resin. Although the CFRP has high strength, it cannot effectively improve the stiffness of the reinforced structures because the thickness of CFRP is thin and less than 1 mm. Moreover, the price of CFRP is high. 

To overcome the shortcoming of conventional CFRP plate, a composite strengthened steel-carbon fiber reinforced polymer (SCFRP) is proposed in this paper, in which a kind of high-strength steel wire is integrated into the carbon fiber plate [26,27]. In the manufacturing process, part of the carbon fiber silks was replaced by high-strength steel wires, and the steel wires are completely wrapped with carbon fiber silks. Under the tension of external force, the carbon fiber silks and the steel wires are molded to produce SCFRP. Compared with conventional CFRP, SCFRP has the advantage of higher strength and cost-effectiveness.

In this study, SCFRP with 7.8% steel wires, is used for prestressing and the properties of SCFRP are listed in Table 2. The width and thickness of SCFRP are 50 mm and 3mm, respectively.

### 3.2. Prestress Strengthening

**Figure 3:**
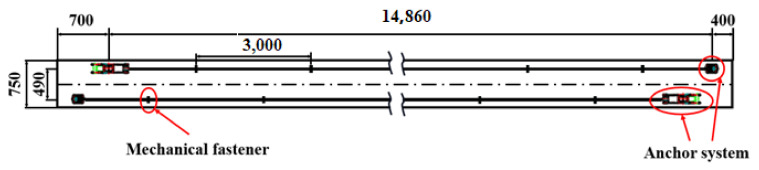
Prestressed SCFRP on the bottom of damaged RC beam.

An experimental study was carried out on a full-size RC beam strengthened with prestressed SCFRP in field. In the test, three prestressing levels were designed for SCFRP: *f*_p1_ = 720 MPa (30% of *f*_pu_), *f*_p2_ = 960 MPa (40% of *f*_pu_), and *f*_p3_ = 1440 MPa (60% of *f*_pu_). Specimens A2P30, A3P40, and A4P60 were damaged RC beams strengthened by SCFRP with 30%, 40%, and 60% prestressing level, respectively. The schematic of prestressing SCFRP is shown in Figure 3, and the strengthening proceeded as follows.

Before strengthening with prestressed SCFRP, the visible cracks with more than 2 mm width on the side and bottom of damaged RC beams were repaired by glue. To improve the bond performance, the bottom surface of the damaged RC beam was repaired with primer and putty.

**Figure 4:**
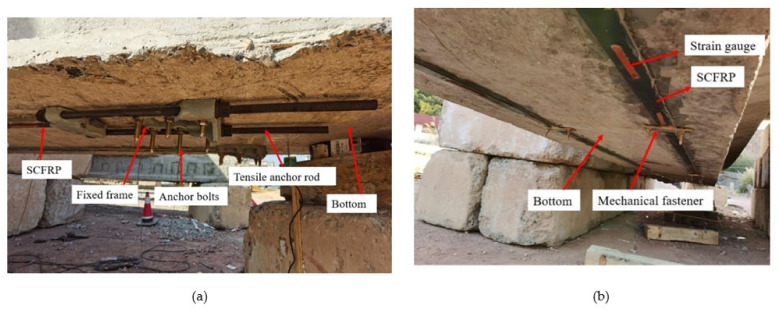
The site of anchor system: (**a**) anchor system; (**b**) prestressed SCFRP.

An adequate anchor system was designed to avoid the peeling of SCFRP. Before prestressing, the anchors were bolted to the bottom of the damaged RC beam end, as shown in Figure 4a.

The SCFRP plates were installed on the anchor system, and the two ends were connected with the horizontal counterforce frame through tension rods. One end of SCFRP is fixed to anchor, and the other end is loaded by external force.

During the process of prestressing, the axis of the SCFRP remained at the same level under the external force. The elongation of SCFRP is calculated by the elongation of the jack and shrinkage of the anchor. Multi-stage loading was adopted and SCFRP were therefore prestressed.

As in the filed application, the tensile loads were applied and sustained for 15 min to keep the expected prestressing level. After prestressing, the epoxy resin was filled between the SCFRP and concrete. Moreover, the prestressed SCFRP were bonded to the concrete.

To reduce the debonding failure on SCFRP-concrete interface, four mechanical fasteners were installed on each SCFRP, as shown in Figure 4b. 

### 3.3. Test Setup in Field Condition

**Figure 5:**
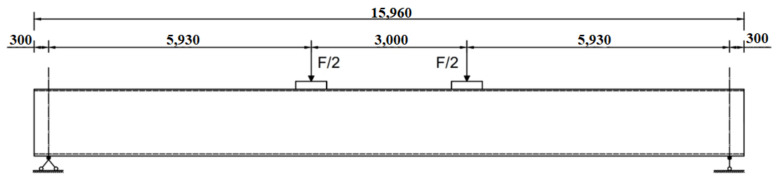
Four-point bending test (all the dimensions are in mm).

**Figure 6:**
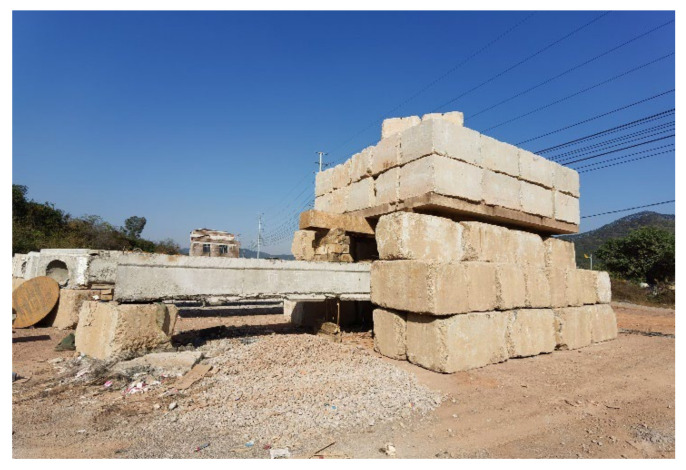
Field condition.

**Figure 7:**
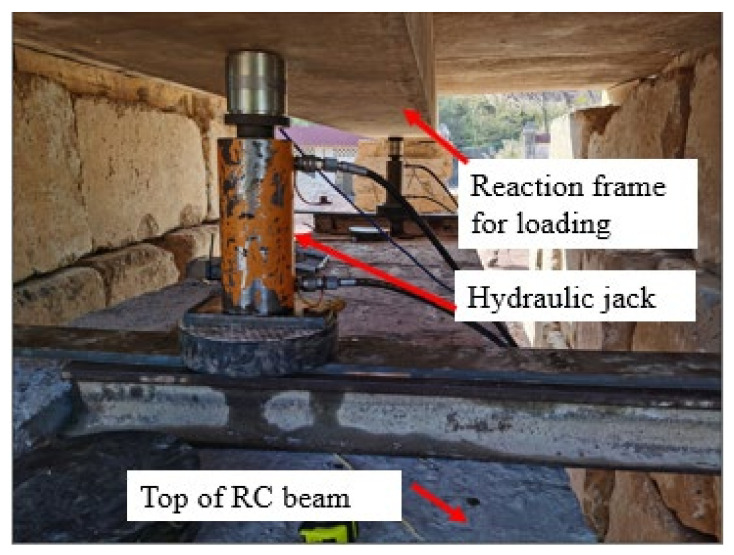
Loading device.

As shown in Figure 5, the damaged RC beams were performed in four-point bending across a span of 15.36 m, and they were loaded in field condition through hydraulic jacks acting on rigid counter force frame consisting of steel beam and huge stones. Moreover, the loading value was acquired by static data acquisition instrument connected with the loading sensor. The view of field condition and loading device are also shown in Figure 6 and Figure 7. 

Before testing, some visible cracks with more than 2 mm width were repaired by glue. Moreover, gratings were plotted for available observation of crack propagation. The crack width can be measured by digital concrete crack width gauge. To obtain the tensile stress of the steel reinforcement during the test, the steel reinforcement was exposed at the middle bottom of RC beam after the concrete was removed. One strain gauge was bonded on steel reinforcement with epoxy resin and connected with GBD3816 static data acquisition instrument. Meanwhile, a total of 40 strain gauges are directly attached on the concrete surface of the damaged RC beam along the length, 12 of which were attached at the middle span of the top and bottom of RC beam, 8 on the left and right side of the web symmetrically. All the strain gauges were connected with GBD3816 static data acquisition instrument. To measure the deflections of damaged RC beam, 14 extensometers were placed symmetrically in 7 control points, including two supports, two quarter spans, two loading points and mid-span. Each control points arranged 2 extensometers. Fourteen extensometers were also connected with GBD3816 static data acquisition instrument. 

The test was carried out based on standard [28] and the damaged beam was preloaded first. Loading step was F/2=30 kN. During the process, the maximum crack width, the stress of concrete on bottom, web and top plate, the tensile strain of main steel reinforcements, and deflection of damaged RC beam were measured.

## 4. Results and Discussion

### 4.1. Validation of Capacity and Damage Evaluation 

**Figure 8:**
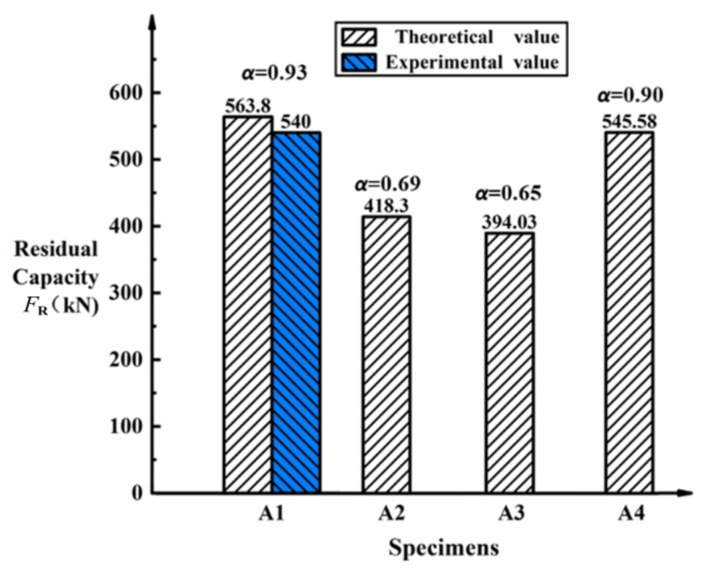
The details of residual capacity.

In Section 2.2 and Section 2.3, the capacity of hollow RC beam without damage and with damage evaluation were analyzed. Substituting the geometrical and material parameters into equation (2), the theoretical value of the flexural load capacity *M*_u_ of hollow RC beam without damage is 614 kN·m and the four-point bending ultimate load is 606.2 kN. Meanwhile, after considering the damage evaluation, the strength reduction coefficient α of A_1_ and theoretical value of residual capacity *F*_R_ are 0.93 and 563.8 kN, respectively. Compared to experimental results of 540 kN, the error is 4.4%. It showed that the calculated results of capacity of hollow RC beam without damage and damage evaluation are in good agreement with experimental results. For the four-point bending described in Figure 5, the theoretical values of residual capacity *F*_R_ of specimens A2, A3, and A4 are 418.30, 394.03, and 545.58 kN, respectively. The details of residual capacity of each damaged RC beam before strengthening are shown in Figure 8.

### 4.2. Stiffness

**Figure 9:**
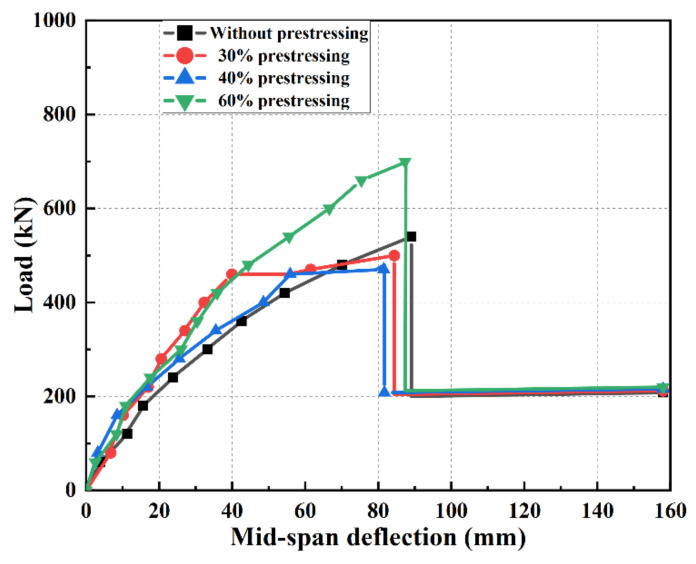
Load-deflection curves of all specimens.

During the tests, the mid-span deflections of four specimens were monitored. The curves of four specimens with the applied load and mid-span deflection are shown in Figure 9. The details of all specimens and the important parameters for charactering the behavior of damaged hollow RC beams with and without strengthened by prestressing SCFRP are summarized in Table 3. 

As expected, at the same damage level, the flexural behavior of damaged RC beam strengthened by SCFRP with 60% prestressing level (A4P60) was significantly improved compared to that of specimen A1. Moreover, the yield load and ultimate load were increased by 25% and 33.3%, respectively. The results showed the prestressing force can reduce the tensile force of steel reinforcements and especially enhance the yield load.

With the same damage level and similar prestressing strengthen, the load versus mid-span deflection curves of specimens A2P30 and A3P40 were also similar. As shown in Figure 9, both had clear inflection points. After the inflection points, the mid-span deflection increased rapidly. Compared to specimen A1, the severely damaged specimens can be recovered to undamaged state after being strengthened by prestressed SCFRP. Moreover, the cracking load of specimens strengthened by prestressed is higher than that of A1.

The stiffness at the elastic stage and the cracked stage were denoted by *K*_I_ and *K*_II_, and the values are also listed in Table 3. It can be seen from the Table 3 and Figure 9 that *K*_I_ of prestressed specimens increased significantly. The result showed the prestress can effectively improve the stiffness of RC beams before cracking. However, *K*_II_ did not show a similar trend as *K*_I_, and the change is not obvious after cracking. Meanwhile, for all specimens, the ultimate deflections were almost same. 

### 4.3. Failure Modes

**Figure 10:**
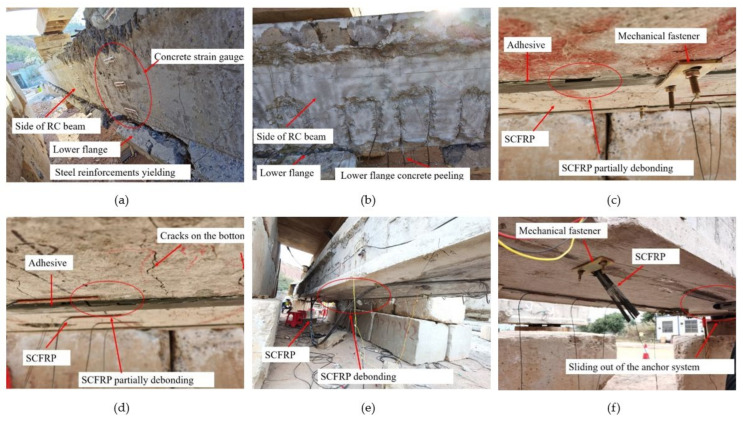
Failure modes: (**a**) steel reinforcement yielding; (**b**) concrete peeling; (**c**,**d**) SCFRP partially debonding; (**e**) SCFRP debonding; (**f**) sliding out.

The failure mode of the validate specimen A1 and A2P30 is SY (flexural failure with steel reinforcements yielding), followed by lower flange concrete peeling and upper concrete crushing, as shown in Figure 10a. For specimen A2P30, the failure mode of CP (lower flange concrete peeling) is more serious, as shown in Figure 10b.

The specimen A3P40, strengthened by SCFRP with 40% prestressing level, presented flexural failure caused by steel reinforcements yielding (SY) and plate partially debonding (PD), as shown in Figure 10c,d. 

Specimen A4P60 failed by SCFRP debonding (DB) and sliding out of the anchor system (PS), as shown in Figure 10e, and the failure of SCFRP fracture also occurred, as shown in Figure 10f. 

It was observed that the debonding failure is more likely to occur with the increasing of plate prestressing level. When the prestressing level increased to 40% and 60%, the apparent debonding failure occurred, especially at 60% prestressing level. The mechanical fastener can reduce the debonding failure when the prestressing level is 30%. 

### 4.4. Strain Development

#### 4.4.1. Load–Strain Curves of Steel Reinforcement

**Figure 11:**
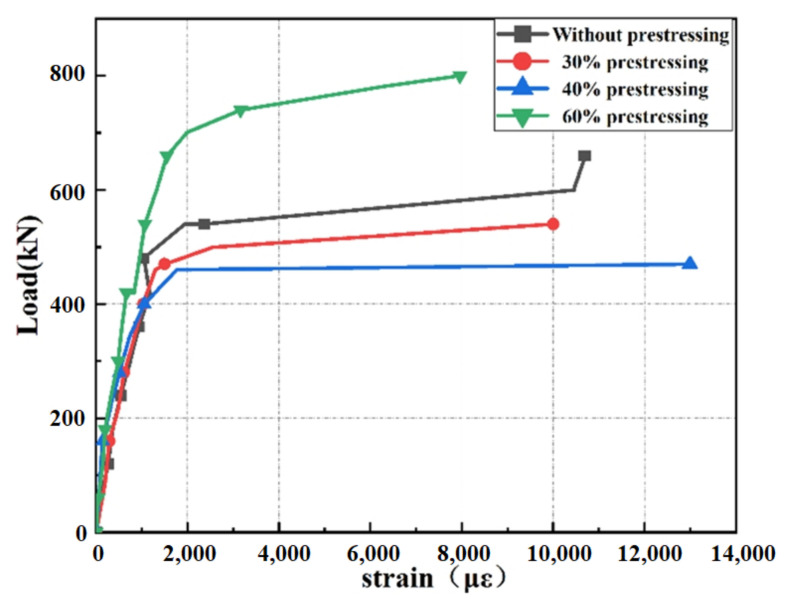
Load versus steel reinforcement strain curve.

The load–strain curves of steel reinforcement are shown in Figure 11. It can be seen that the strain of steel reinforcement increased slowly before yielding. With the same damage level, the prestressed SCFRP can effectively reduce the strain of steel reinforcement of specimen A4P60 compared with that of A1. The yield load of specimen A4P60 was 240 kN, which increased by 71.4%. When the steel reinforcement reached the tensile strength, the corresponding loads of A1 and A4P60 were 540 and 720 kN, respectively. The ultimate load of A4P60 increased by 33.3% due to the presence of prestressed SCFRP. 

With the same damage level, the yield loads of specimens A2P30 and A3P40 were 200 kN and 220 kN after being strengthened by SCFRP with 30% and 40% prestressing level. When the steel reinforcement reached the tensile strength, the corresponding loads of two specimens were 500 and 480 kN, respectively. Compared with that of A1, the flexural behavior of badly damaged specimens can be recovered after being strengthened by prestressed SCFRP.

Based on the analysis of capacity and damage evaluation mentioned in Section 2, the ultimate loads of A2P30 and A3P40 were 363 and 342 kN before strengthening, but increased to 500 and 480 kN after being strengthened. The increments were 37.7% and 40.4%, respectively.

#### 4.4.2. Load–Strain Curves of Concrete 

**Figure 12:**
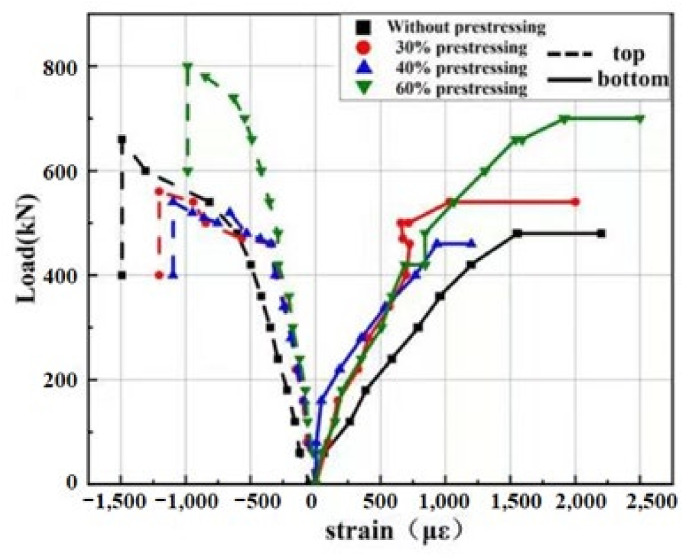
Load versus concrete strain curve.

In the experiment, the concrete strains placed at the middle span of the top and bottom plate were recorded and the curves are presented in Figure 12. As for concrete strain on top beam, the compressive strain of specimens A4P60 was the smallest among the four specimens. When the specimen A4P60 reached failure, the compressive strain was 990 με. On the contrary, the concrete strain of beam A1 without strengthening was the largest when the specimen was fractured, and the compressive strain was 1492 με. The load versus concrete compression strain curves of A2P30 and A3P40 were similar, and the two specimens failed when the compressive strains were 1205 με and 1098 με, respectively. It can be seen from the results that the action of prestressed SCFRP can effectively reduce the concrete compressive strain on top beam, which was consistent with the experimental phenomenon of partial concrete crushing in specimen A1. Moreover, there was one load inflection point in all curves of load versus concrete compressive strain, which corresponded to the load when the steel reinforcement reached the tensile strength.

The curves of load versus concrete tensile strain on bottom of mid-span specimen are also illustrated in Figure 12. It can be seen that under the same load level, the concrete tensile strain of specimen A4P60 strengthened by 60% prestress level was the smallest, while that of specimen A1 without strengthening was the largest. The results show that the prestress efficiently improved the tensile stress of concrete and reduced the possibility of early cracking initiation. However, there was also one load inflection point in all curves, as shown in Figure 12, which was mainly caused by the concrete cracking at the bottom of the specimen. The corresponding cracking loads of four specimens are 120, 180, 140, and 190 kN, respectively.

#### 4.4.3. Load–Strain Curves of SCFRP 

**Figure 13:**
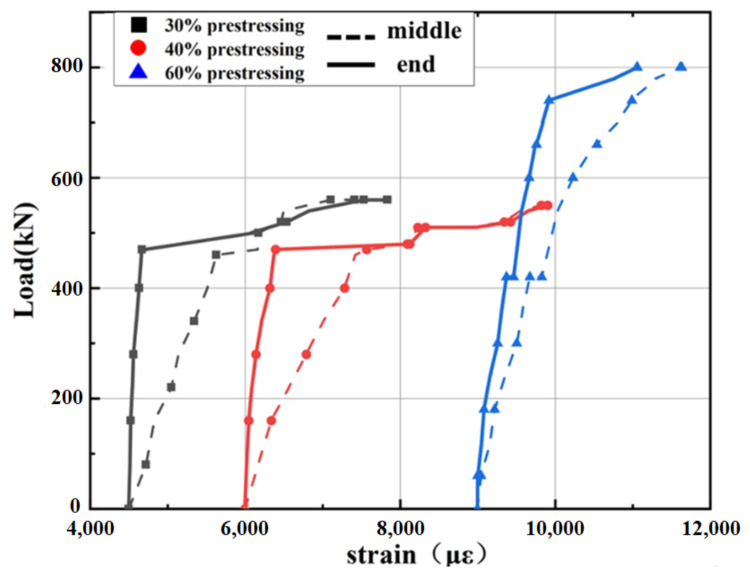
Load versus SCFRP strain curve.

The strains located on middle and end of SCFRP were measured, and the curves of load versus SCFRP strain are plotted in Figure 13. It can be observed that the curves of the three prestressed specimens had similar characteristics, which are summarized as follows: (1) With the increase of load, the strains at the middle of the SCFRP increased rapidly, while the strains at the end of the anchorage increased slowly. However, when the load reached the inflection point, the strains at the end of the anchorage improved sharply and tended to be consistent with the strain at the middle of the SCFRP. The phenomenon was caused by the large deformation at the middle of span during the loading process. When the steel reinforcement reached the ultimate strength, the prestressed SCFRP were subjected to main external load, and the force in mid-span was transferred to SCFRP near the anchorage, leading to the strains increasing sharply and tending to be consistent along the whole SCFRP; (2) the strain curve at the end of the anchorage had one load inflection point, which corresponded to the load when the steel reinforcement reached the ultimate tensile strength. However, there were two load inflection points in the strain curve at the middle of the SCFRP, which corresponded to the cracking load and the load when the steel reinforcement reached its tensile strength.

There are some differences among all the prestressing SCFRP-strengthened specimens. The curves for specimens with 30% and 40% prestressing level (A2P30 and A3P40) show the same trend, that is when the steel reinforcement reached maximum bearing capacity, the strains of SFRP increased sharply but the load did not increase. However, specimen with 60% prestressing level (A4P60) had a different trend of load-SCFRP strain curve, in which SCFRP strain increased slightly and then showed the brittle failure character. In conclusion, it would be detrimental for the strengthened structure to render SCFRP a high prestressing level, especially more than 60%. 

#### 4.4.4. Concrete Strain Distribution over the Depth

**Figure 14:**
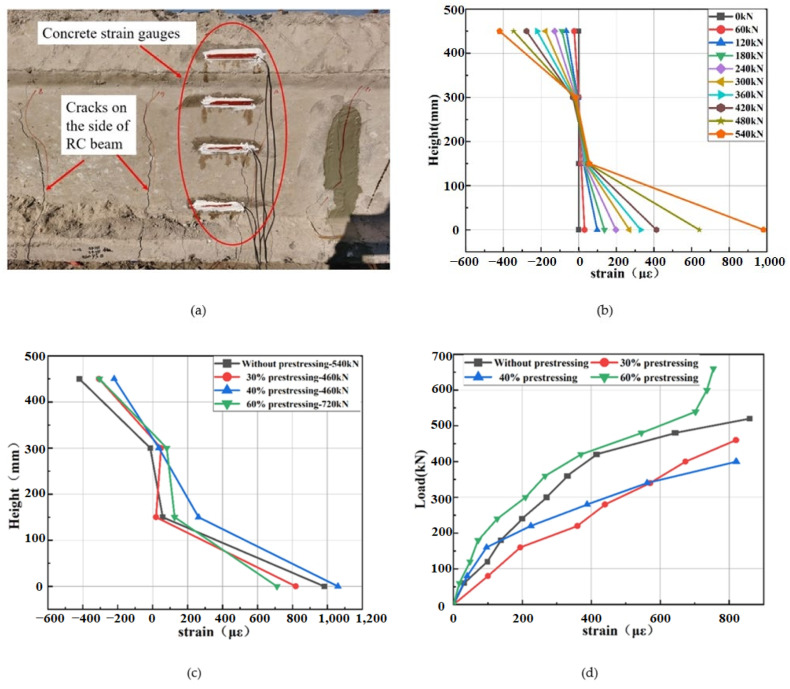
The concrete distribution over the depth of the specimens: (**a**) strain gauges distribution; (**b**) the value of concrete strain; (**c**) concrete strains under ultimate load; (**d**) the mid-span tensile stress at the bottom of concrete.

To obtain the strain distribution law in concrete over the depth of RC beams, four strain gauges were bonded on the mid-span of RC beam, as shown in Figure 14a. Figure 14b presents the strain value of concrete under different loading level. During the elastic stage at the beginning of loading, the strain distribution was linear. Under the ultimate load, the distribution of concrete strains on the sides of the four specimens is illustrated in Figure 14c. It can be found that the concrete tensile stress at the bottom of RC beam strengthened by 60% prestressing level was the smallest. The prestressing method can reduce the tensile stress of concrete at the bottom of the structure. The severely damaged specimens A2P30 and A3P40 can almost be recovered under the action of prestressed SCFRP. In the ultimate load condition, the concrete compressive stress at the top of the unreinforced RC beam was the largest compared to the prestressing strengthened specimen. With the increase of prestressing level, the neutral axis height went up. 

The mid-span tensile stress at the bottom of concrete during the whole loading process is shown in Figure 14d. The load corresponding to the inflection point in the early stage was the cracking load. It can be observed that the cracking load of the unreinforced beam was the smallest, while that of 60% prestressing level specimen was the largest. This indicated that prestressing can reduce the concrete cracking effectively. 

### 4.5. Crack Development

**Figure 15:**
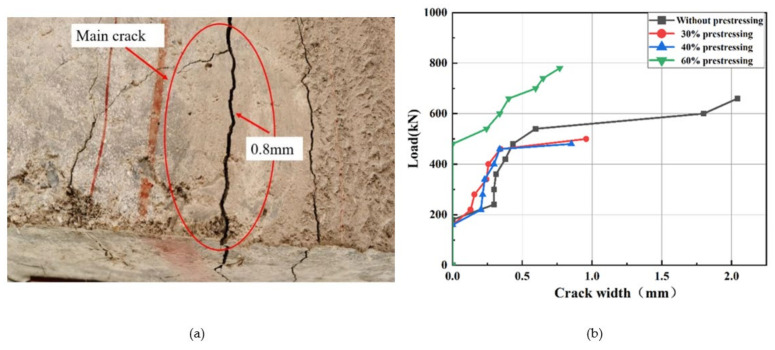
Main crack development: (**a**) main crack pattern; (**b**) main crack width versus load curves.

During the test, the crack width was measured, as shown in Figure 15a. The main crack propagation law in the mid-span of specimens is illustrated in Figure 15b. It can be seen from the figure that these curves have the similar trend. Under the same loading level, the width of main crack of the unreinforced specimen was evidently larger than that of prestressing-strengthened specimens. The result indicates that prestressing can reduce crack propagation effectively. In addition to new cracks, the cracks repaired by grouting initialed again during loading.

During the loading process, the crack distribution was observed. Compared with the unreinforced specimen, the crack width propagation law of prestress-strengthened specimen was smaller, and the crack distribution was more dense.

## 5. Conclusions

In this paper, field experiments with full-scale damaged hollow RC beams were carried out to study the flexural behavior of such damaged beams strengthened with prestressed SCFRP, which is a new kind of high-strength material consisting of steel wires and carbon fiber silks. Before the strengthening process, the damage level of each specimen was evaluated based on the codes. Through the four-point bending experiments, the failure modes, deflection, yield and ultimate load, strains of concrete, steel reinforcements, and SCFRP were measured and analyzed. From the test results, several conclusions were obtained as follow:

The effectiveness of prestressing SCFRP was evaluated considering the damaged levels of aged full-scale hollow RC beams.

The stiffness at the elastic stage was increased by 64.9%, 66.9%, and 67.1% after strengthened by SCFRP with 30%, 40%, and 60% prestressing level, respectively.

Considering the damage, the cracking load, yield load, and ultimate load of damaged hollow RC beam are obviously increased because of the presence of prestressed SCFRP. As the full-scale damaged hollow RC beams were strengthened by 30%, 40%, and 60% prestressing level of SCFRP, the strength increases 19.53%, 21.82%, and 31.9%, respectively.

Prestressing can effectively control the crack development. The higher the prestressing level, the smaller the crack width. 

The failure mode of the damaged beams strengthened with high prestressing level is SCFRP-concrete interface debonding. The high prestress level is more likely to cause interfacial debonding failure.

The flexural behavior of the severely damaged RC beam with strength reduction coefficient of 0.65 can be recovered after being strengthened by SCFR with 40% prestressing level.

Based on the strains of steel reinforcement and SCFRP during loading, the characteristics of brittle failure became more evident with the increased prestressing level of the SCFRP. It would be detrimental for the strengthened structure strengthened by SCFRP with a high prestressing level, especially more than 60% of ultimate strength.

## Figures and Tables

**Table 1:** Damage evaluation of four damaged RC beam.

Damaged RC Beam	Cross-Section Reduction Factor *R*	Structural Detection Factor Z	Deterioration Factor $\zeta_{e}$	Strength Reduction Coefficient α	Flexural Load Capacity *M*u (kN·m)
A1	0.96	0.98	0.011	0.93	1501.02
A2	0.85	0.89	0.083	0.69	1113.66
A3	0.80	0.89	0.085	0.65	1049.1
A4	0.95	0.96	0.01	0.90	1452.6

**Table 2:** Properties of SCFRP [26].

Types	Tensile Strength *f*_pu_ (MPa)	Elastic Modulus *E*_p_ (GPa)	Elongation
SCFRP	2400	160.1	1.5%

**Table 3:** Details of specimens and summary of test results.

Specimen No.	Strengthening Method	Stiffness		Cracking		Yielding		Ultimate		Failure Modes	Increased Ratio (%) (*F_u_* − *F_R_*)/*F_R_*
		*K*_I_	*K*_II_	*F_cr_* (kN)	*δ_cr_* (mm)	*F_y_* (kN)	*δ_y_* (mm)	*F_u_* (kN)	*δ_u_* (mm)		
A1	without strengthening	10.62	6.12	120	11.3	480	70.1	540	89.1	SY, CP	--
A2P30	30% prestressing	16.49	5.99	160	9.7	470	61.45	500	84.4	SY, CP, CC	19.53
A3P40	40% prestressing	17.72	6.71	140	7.9	440	52.6	480	81.7	SY, PD, CC	21.82
A4P60	60% prestressing	17.75	7.34	190	10.7	600	66.5	720	87.4	DB, PS, PF, CC	31.9

Note: SY refers to the flexural failure caused by steel reinforcements yielding; CP refers to the lower flange concrete peeling; CC refers to the concrete crushing; PD refers to the SCFRP partially debonding; DB refers to the SCFRP debonding failure; PS refers to the SCFRP sliding out of the anchor system; PF is the SCFRP fracture.

## Data Availability

Data are available from the authors upon request.
